# Experimental Study on Self Compacting Fibrous Concrete Comprising Magnesium Sulphate Solution Treated Recycled Aggregates

**DOI:** 10.3390/ma15010340

**Published:** 2022-01-04

**Authors:** Parthiban Kathirvel, Gunasekaran Murali, Nikolai Ivanovich Vatin, Sallal R. Abid

**Affiliations:** 1 School of Civil Engineering, SASTRA Deemed University, Thanjavur 613404, India; 2 Peter the Great St. Petersburg Polytechnic University, 195251 St. Petersburg, Russia; vatin@mail.ru; 3 Civil Engineering Department, Wasit University, Kut 52003, Iraq; sallal@uowasit.edu.iq

**Keywords:** recycled aggregate, sustainable treatment, magnesium sulphate self-compacting concrete, strength

## Abstract

It appears that the awareness and intentions to use recycled concrete aggregate (RCA) in concrete are expanding over the globe. The production of self-compacting concrete (SCC) using RCA is an emerging field in the construction sector. However, the highly porous and absorptive nature of adhered mortar on RCA’s surface leads to reduced concrete strength, which can be removed with the application of various techniques, such as acid treatment. This study investigated the effect of the partial replacement of silica fume by cement and natural aggregate (NA) by RCA with and without steel fibre. The used RCA was treated with magnesium sulphate solution. It was immersed in solutions with different concentrations of 10%, 15% and 20% and for different periods of 5, 10 and 15 days. Sixteen mixes were prepared, which were divided into six groups with or without 1% of steel fibre content. The fresh properties, compressive strength, split tensile strength and impact resistance were examined. The results revealed that the strengths of the mixes with 20% RCA were marginally better than those of the control mixes. The compressive strength and split tensile strength were reduced by 34% and 35% at 60% RCA content, respectively, as compared to the control mixes.

## 1. Introduction

The fundamental environmental issues related to cement production are the intake of raw materials, power consumption and emissions of air pollutants. In the last two decades, global cement production has tripled from 1.10 to 3.27 billion tonnes [1,2]. It is anticipated that by 2030, global cement production will reach 4.83 billion tonnes [1,2]. The use of industrial by products such as ground granulated blast furnace slag (GGBFS), silica fume and fly ash can significantly reduce PC utilisation in concrete production [3,4,5,6]. Next to cement, the aggregate occupies a significant concrete component, the quarrying of which has been projected to reach 51.7 billion metric tonnes per year from 48.3 billion metric tonnes per year on average 2015–2019, representing an annual growth rate of 5.2%. The practical application of recycled concrete aggregate (RCA) in the building sector has attracted the attention of many in the green and resource management communities [7]. However, the influence of RCA has often been less significant than that of natural aggregates (NA) because of the adhered mortar that is present on the surface of RCA, which results in high absorption and porosity [8]. Talamona and Tan [9] stated that as the amount of RCA replacing NA increases, the compressive strength of the concrete decreases significantly.

According to earlier studies, mechanical parameters of elastic modulus, tensile strength and compressive strength might be attained using RCA in structural applications [10,11]. Poon et al. [12] reported that if NA is partly or entirely replaced with RCA, the splitting tensile strength declines by 10–24% and the compressive strength declines by 12–30% [13]. Earlier studies [14,15] reported that the RAC concrete’s critical strain increased 10–20%, while its elastic modulus decreased 20–25%. According to Qing et al. [16], RCA may diminish concrete deterioration when the replacement rate is low; however, when the replacement rate is increased, concrete deterioration increases. Behera et al. [17] stated that using 100% RCA in place of virgin aggregate decreases the compressive strength by 30 to 40%. According to the results of the experimental investigation of Rao et al. [18], the reduction in splitting tensile strength may reach up to 24% when RCA is used to fully replace the NA. The quality and surface properties of RCA, rather than the replacement amount of RCA, have been recognised as the most important factors influencing the flexural and splitting tensile strength of recycled aggregate concrete (RAC) in most instances. Zuhud [19] stated that the bond strength between concrete and deformed bars was improved as the proportion of RCA in the concrete increased. The 30% and 60% RCA replacements led to 32.4% and 46.1% higher bond strength than conventional concrete. Limbachiya et al. [20] reported that concrete with greater RCA content was more susceptible to sulphate attack. Better resistance to sulphuric acid attack was found when fly ash was included in RAC rather than the standard Portland cement. RAC with 100% RCA demonstrated a 73.2% increase in chloride conductivity at the end of 28 days [21]. Lu et al. [22] reported that the water absorption of RCAs with particle sizes of 5–10 mm and 10–20 mm was decreased by up to 30% and 22% following rapid carbonation treatment, and the apparent density could be improved by up to 4.8% and 3.2%, respectively.

With the intention of condensing the absorption characteristics of RCA, diverse treatment techniques have been used—namely, ultrasonic cleaning method [23], heating and subsequent rubbing [24,25], ball milling [26], etc. The development of RCA characteristics has been attained by surface coating with water glass [27] and polyvinyl alcohol emulsion [28]. Soaking RCA in acid solutions such as HCl, H_2_SO_4_ and H_3_PO_4_ causes a significant reduction in the absorption characteristics and augmented mechanical characteristics [29]. The effects of HNO_3_ [30] and CH_3_COOH [31] were also investigated to eliminate the deposit of adhered mortar from RCA. While all these treatment techniques are viable, they have their drawbacks. For instance, the acid treatment method leads to an increase in the aggregate’s pH, subsequently generating environmental disorder while being disposed to open landfills. With reference to polymer emulsion treatment [28], the hydration progression barrier has been perceived, since the polymer makes the aggregate hydrophobic. Hence, this investigation has been carried out to study the influence of treating RCA with a magnesium sulphate solution, the disposal of which would not create any environmental issues. External sulphate can break the bonds among cement mortar and aggregate by shaping a sulphoaluminate compound—namely, ettringite (3CaO∙Al_2_O_3_∙3CaSO_4_∙32H_2_O)—in the cement. Within the first few hours after mixing with water, the external sulphate reacts with gypsum and calcium aluminate to form ettringite. As a consequence of forming ettringite, the bonding between mortar and aggregate is weakened. The effect of sulphate solutions containing magnesium is more vigorous than those without magnesium, since magnesium reacts with calcium to form brucite and magnesium silicate hydrates.

Many studies have shown that RCAs can be used to produce SCC. To achieve this, the water–cement ratio, the appropriate utilisation of chemical and mineral admixtures and the necessary preparation processes before utilising RCAs must all be controlled [32,33]. Silica fume, an air-entraining agent and a pre-soaking procedure for preparing RCAs reduced the bleeding and segregation of SCC that included fine and coarse RCAs. This phenomenon is due to the small particles of silica fume, which may effectively regulate the concrete’s bleeding and segregation [33]. Al-Kheetan et al. [34] reported that increasing the amount of superplasticizer in the SCC lowers surface voids, which in turn limits the penetration of impregnants, resulting in a slightly greater chloride penetration. Gesoglu et al. [35] reported that the combined utilisation of fine and coarse RCA in SCC with 100% replacement decreases the compressive strength by up to 31% and reduces the bending strength, modulus of elasticity and tensile strength. However, the incorporation of silica fume enhances the mechanical properties of these mixes. Ahmadi et al. [36] investigated the compressive strength of SSC mixes prepared with rise husk ash of 10% and 20% concentrations with two different water to cement ratios of 0.35 and 0.4 for a total of 180 days. SCC mixes outperformed conventional concrete in terms of compressive strength by 31–41%. Abid et al. [37] reported that the micro-steel fibres significantly improved the impact resistance and ductility of the SCC specimens. There was a noticeable difference in the enhancement at the failure stage compared to the initial crack stage. The greatest attained improvements were 543% and 836%, respectively, at the cracking and failure stages for the 30 MPa mixes.

This investigation evaluates the influence of MgSO_4_ concentration and the process duration on the treatment of RCA. From the superior physical properties, RCA treated with the optimum dosage and process duration were taken for the following investigation stage, where the fresh and hardened characteristics of SCC were investigated considering the partial replacement of NA by RCA (0%, 20%, 40% and 60%) and cement by silica fume (0%, 10% and 20%), and the inclusion of steel fibres was the third investigated parameter. The SCC mixes fresh properties were estimated with the aid of density, slump flow, T_500_, J-ring and V-funnel test. The investigated hardened properties were the compressive strength, split tensile strength and drop-weight impact resistance.

## 2. Materials and Methods

### 2.1. Materials

Grade 53 Ordinary Portland Cement (OPC) confirming to IS 12269 [38] having a fineness of 340 m^2^/kg, initial setting time of 38 min, soundness of 3 mm, specific gravity of 3.13 and standard consistency of 28% was utilised in this investigation. Ground granulated blast furnace slag (GGBFS) was utilised as a supplementary cementing material (SCM) to achieve an enhanced strength and durability characteristics of hardened concrete. GGBFS is illustrated by superior strength, reduced hydration heat, resistance to chemical attack, enhanced workability, superior durability characteristics and cost-effectiveness. The fineness and specific gravity of the GGBFS used in this investigation were 380 m^2^/kg and 2.80, respectively. The chemical composition of OPC and GGBFS are detailed in Table 1.

**Figure 1:**
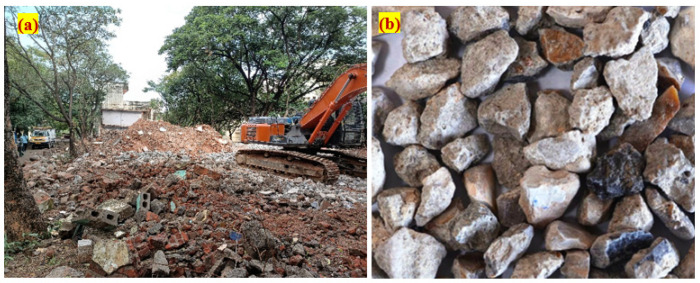
Details of RCA: (**a**) source (**b**) appearance of recycled aggregate.

Fine aggregate of manufactured sand (M-sand) type was used in this investigation with a specific gravity 2.62 and bulk density 1774 kg/m^3^, which was used as an alternate river sand. Coarse aggregate of crushed granite type derived from the natural disintegration of rock, crushing of hard stone/gravel available in a local quarry and crushed blue granite stones was used in this investigation as natural coarse aggregate (NA). The physical properties of the sample used in this investigation were: abrasion resistance—17.8%, impact resistance—15.4%, crushing strength—28.7%, water absorption—0.77%, bulk density—1478 kg/m^3^, fineness modulus—6.67, specific gravity—2.73. Recycled concrete aggregate derived from the demolished concrete waste from SASTRA University was also utilised in this study as RCA to partially replace NA. The source of recycled aggregate and appearance of derived recycled aggregate are shown in Figure 1.

**Figure 2:**
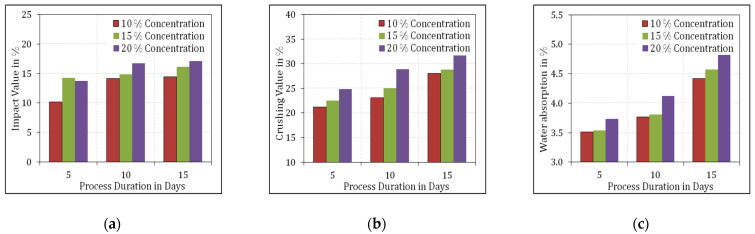
Physical properties of treated RCA samples. (**a**) Impact value, (**b**) Crushing value, (**c**) Water absorption.

Initially, the aggregates resulting from the recycled concrete acquired from C&D waste was soaked in water to get rid of the disproportionate chloride substance, as suggested by Debieb et al. [39]. The aggregates’ drying was done under air and not by oven drying to prevent any possible superior premature slump and speedy loss in slump [40]. Magnesium sulphate crystals were fetched and MgSO_4_ solution was prepared with distilled water in 3 different concentrations of 10%, 15% and 20%. The crushed RCA was immersed in the MgSO_4_ solution for 3 process periods of 5, 10 and 15 days. After the respective process periods, the RCA was washed with tap water to chuck out the enduring MgSO_4_ and finally was desiccated in atmospheric temperature. The physical characteristics of the treated aggregates were investigated as per IS 2386 [41] and their results are projected in Figure 2.

**Figure 3:**
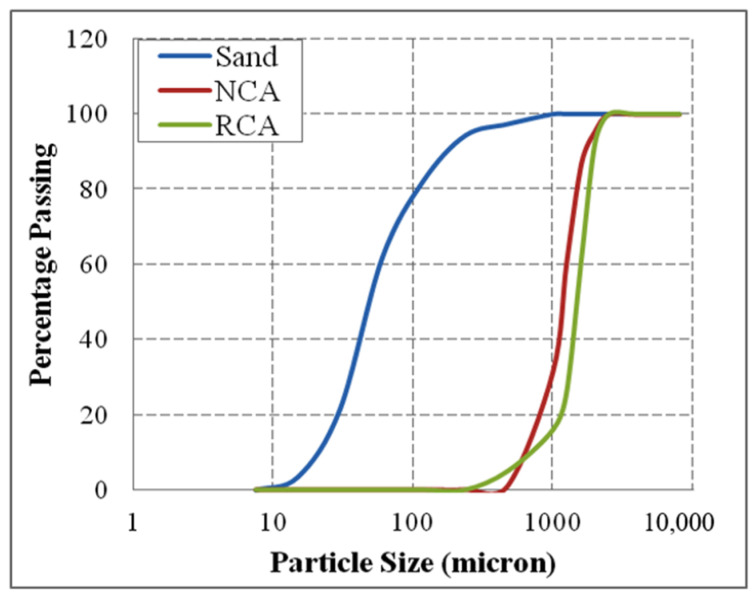
Particle size distribution in the aggregates sample.

From Figure 2, it is clear that the physical properties of the treated RCA were found to be inferior with the increase in the concentration of MgSO_4_ and the process duration. Hence, the subsequent investigation on the application of RCA in evaluating the fresh and hardened properties of SCC was carried out with the RCA treated with 10% MgSO_4_ concentration over a process duration of 5 days. The particle size distribution of the sample of aggregates used in this investigation is shown in Figure 3.

Steel fibres are used in concrete mixes as short, discrete reinforcing elements to improve ductility, toughness and control the crack propagation. Hooked-end type steel fibres with diameter of 0.75 mm, length of 60 mm and tensile strength of 1225 MPa were used in this investigation. Tec mix-640, a polycarboxylic ether (PCE)-based superplasticizer, was utilised (0.5%) as a chemical admixture. The superplasticizer was light brown liquid with 1.08 ± 0.01 at 25 °C relative density, chloride ion content of less than <0.1% and pH value of 7–9. The purpose of the superplasticizer was to decrease the adverse impact of RCA and steel fibres on the workability of fresh concrete.

### 2.2. Methodology

**Figure 4:**
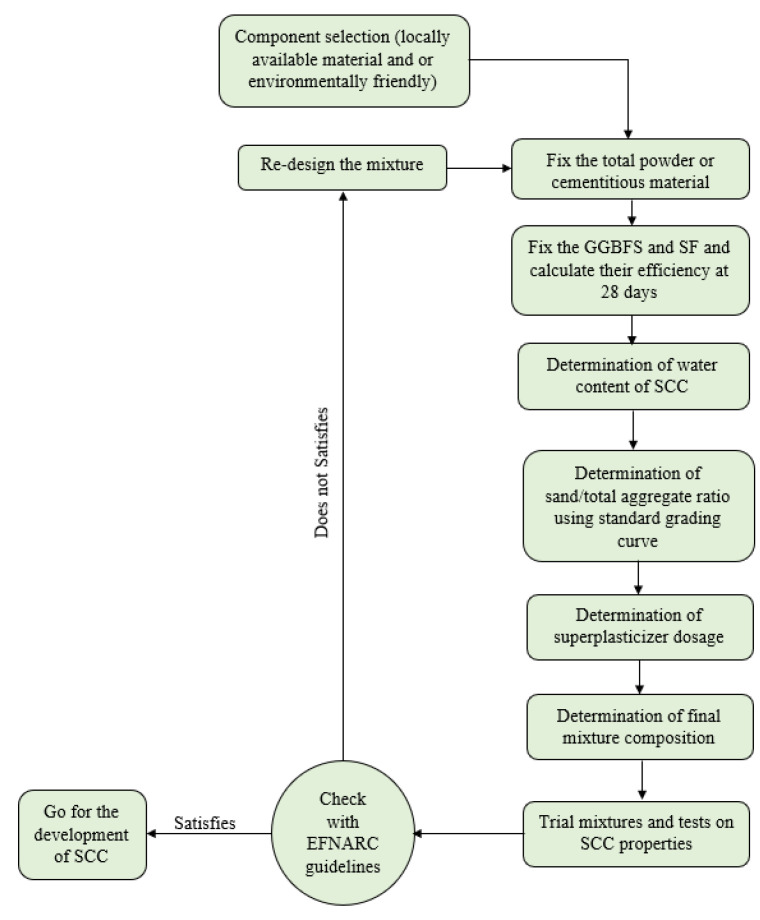
Mix design methodology.

This study investigates the influence of silica fume as a partial replacement of cement (0%, 10% and 20%) and the use of RCA as a partial replacement of NA (0%, 20%, 40% and 60%). The influence of 1% volume fraction of steel fibre was also investigated, where similar mixes were prepared with or without steel fibres. The influence of the study parameters was investigated on the SCC fresh properties and hardened properties at the ages of 7 and 28 days. A fixed partial replacement of 20% of the mix cement by GGBFS was adopted for all mixes. The proportions of the 16 SCC mixes are summarised in Table 2. The outline methodology of designing SCC is shown in Figure 4.

Fresh properties of the developed mixes were assessed using slump flow, T_500_, J-ring and V-funnel tests (self-fabricated in Thanjavur). Inverted slump cone test (self-fabricated in Thanjavur) was used to quantify the concrete flow, which is an indicator for improperly mixed concrete. The cone was first placed in an inverted position on a plate and the concrete mix was filled into the cone through the large opening to reduce spillage. After the concrete had been filled, the cone was lifted up slowly until the concrete mix was totally removed from it and the time taken for concrete to attain the diameter of 550 mm was noted. Similarly, the passing ability of the concrete mix was estimated with the aid of the J-Ring test. It specifies the deformation rate inside an actual flow distance. The cone was lifted up where the concrete flows down the cone, and then the concrete passes through the J-ring. The time required by the concrete to pass through the J-ring was noted, which defines the quality of the concrete. The filling ability of concrete was checked with the application of the V-funnel test. This test is applicable only when the maximum aggregate size in the concrete is 20 mm. Firstly, the V-funnel was filled with the prepared concrete mix and was made to flow down the funnel, where the time taken for the concrete to flow down the apparatus was measured. The recommended values of the developed mixes’ rheological properties as per the guidelines [42] are detailed in Tale 3.

The compressive strength was assessed using 100 mm cubes, and the splitting tensile strength was tested using cylinders 100 mm in diameter and 200 mm in height. The impact resistance was evaluated using cylindrical discs with 150 mm diameter and 63 mm height. The three hardened tests were conducted at two ages of 7 and 28 days. The impact test for the designed mixes was performed by means of a 4.5 kg drop weight that was dropped repeatedly from a height of 457 mm. The process was continued until the cracking and failure of the specimen. The number of blows that are necessary to induce the first crack and failure of the specimen were noted and the corresponding impact energy was computed as given in Equation (1) [43]:

U = N × m × g × h(1)

where U is the impact energy (kN. m or J or N. m), m is the dropping mass (kg), N is the number of retained impact blows, g is the gravitational acceleration (m/s^2^) and h is the drop height (mm).

## 3. Discussion of Results

### 3.1. Fresh Properties

The results are compared with the recommended values of EFNARC guidelines as detailed in Table 3 to check the mixes for their suitability as a SCC. The test results of the rheology properties of the tested mixes are detailed in Table 4. The density of the mixes at fresh state was measured to be 2401 kg/m^3^ for the control mix and the values were found to reduce with increasing the volume of RCA, where the density values ranged between 2317 and 2482 kg/m^3^. This reduction in density might be attributed to the low specific gravity of the RCA as a result of the low-quality adhered mortar on the RCA surface [44]. The variation in density results is compared with the strength results of the hardened mixes in the following sections. The increase in the amount of silica fume was found to increase the density, where silica fume particles act as fine fillers that fill the fine voids and increase the density. The density was also found to be increased with steel fibres addition, which is directly attributed to the much higher density of steel fibres compared to the other ingredients in the mix.

With respect to the flow properties, the slump flow decreased with increasing the volume replacement of NA with RCA and with the inclusion of steel fibres. This behaviour might be attributed to the more porous media of RCA compared to NA due to the adhered mortar, which urges for higher water consumption and hence reduce the workability. On the other hand, the presence of steel fibre is known to hinder the spread and free rolling of mix particles by increasing the internal friction and forming spread obstacles, which negatively impacts the workability of the concrete mix [45]. The slump flow records detailed in Table 4 indicate that the results were in the range of 644–751 mm, which are within the acceptable range of 650–800 mm as per the EFNARC guidelines. However, the slump flow of the mix S6R60F was measured to be 644 mm, which is slightly less than the minimum requirement of 650 mm. The T_500_ results of the tested mixes were found to be within the acceptable range of EFNARC guidelines. The T_500_ records of the introduced mixes were in the range of 2.4–3.7 s. However, the T_500_ value of the mix S6R60F was 5.1 s, which is slightly higher than the EFNARC upper limit of 5 s. The filling and flowability of the developed mixes were estimated in terms of V-funnel apparatus and the results obtained were in the range of 7.8–11.4 s. The results were significantly influenced by the inclusion of steel fibres, silica fume and RCA, where the recorded V-funnel time mostly increased with the increase in RCA and silica fume replacement levels and with the inclusion of steel fibres. However, the results were within the permissible limit of 6–12 s as per EFNARC guidelines for all mixes. The passing ability of the developed mixes was determined with the help of J-ring test and the results were found to be in the range of 7.1–9.0 mm, which are within the acceptable limit of 10 mm as prescribed by EFNARC guidelines. In practice, the mixes with variation in the height below 25 mm are understood to possess an excellent passing ability [46]. Therefore, the results obtained from the developed mixes indicate that the passing ability has been achieved. In general, the workability of the mixes gets affected with the inclusion of RCA, resulting in an increased viscosity and a higher rate of absorption by the RCA, which in most cases cannot be balanced by adding water to the mixes [47].

### 3.2. Compressive Strength

**Figure 5:**
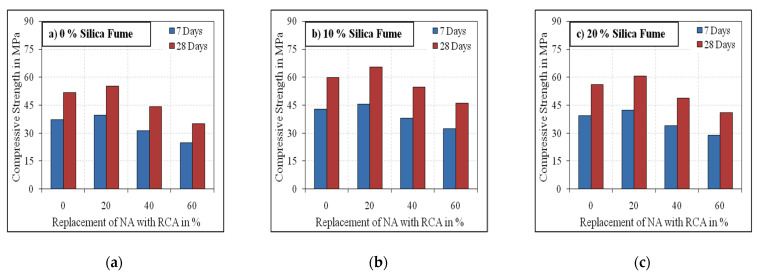
Non-fibrous specimens’ compressive strength. (**a**) 0% Silica fume, (**b**) 10% Silica fume, (**c**) 20% Silica fume.

**Figure 6:**
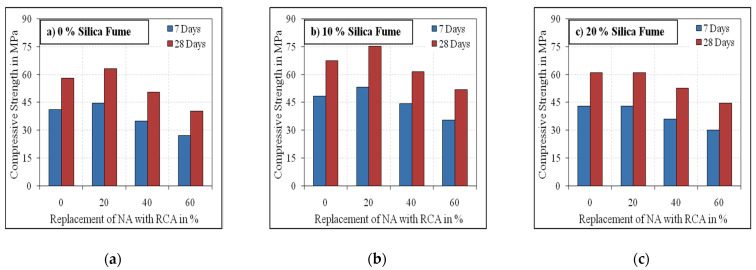
Fibrous specimens’ compressive strength. (**a**) 0% Silica fume, (**b**) 10% Silica fume, (**c**) 20% Silica fume.

Figure 5 and Figure 6 illustrate the compressive strength results of the developed mixes with varying substitution levels of OPC with silica fume and NA and RCA. The effect of the inclusion of steel fibre at the age of 7 and 28 days is also shown in the figures.

It is shown that the compressive strength values at 7 days age ranged from 67.5 to 72% of their corresponding 28 days age values. These values were found to reduce with the increasing volume of RCA beyond 20%. This could be due to the weakness of RCA strength with respect to the natural aggregate and the bond between the RCA surfaces and the new mortar, which is weaker and with double interfacial transition zone (ITZ) to the natural aggregate. A maximum of 11.4% more compressive strength was achieved by the mixes with 20% RCA volume at 28 days curing than the conventional mixes. At 40% and 60% RCA volume, the compressive strength results were observed to be lower by 14% and 31%, respectively, compared to the mixes with NA and 0% silica fume. According to the compressive strength results of the 0% RCA mixes, the mixes containing 10% and 20% silica fume volume were found to be 8.5% to 23% and 13% to 27% higher than the corresponding mixes without silica fume. The findings of the compressive strength tests revealed that the results decreased as the RCA volume increased. However, an improvement in the compressive strength results at 20% RCA volume was recorded for all mix groups and at both ages. A possible explanation for this increase is that the adhered RCA mortar consumes more water than NA, which up to this limit, plays a positive effect on the compressive strength by slightly reducing the actual water-binder ratio compared to conventional concrete [48,49,50]. Whereas at a higher replacement level of NA with RCA, the presence of a high volume of unhydrated adhered mortar on the RCA surfaces would significantly reduce the water content for complete hydration, which results in reduced strength. The reduction in the compressive strength results from the increasing amount of RCA is primarily attributed to the development of fragile interfacial transition zone, the crack formation in RCA and the porosity of the adhered mortar in RCA [51,52,53,54,55,56].

With respect to the effect of silica fume, the 28 days compressive strength results were observed to be in the range of 16 to 32% compared to the mixes without silica fume. However, for the mixes with 20% silica fume, the compressive strength results were observed to be less than the mixes with 10% silica fume, which agrees with results obtained by a previous research [57]. The increase in the compressive strength of mixes with 10% silica fume was irrespective of RCA percent replacement and steel fibre inclusion. This can be attributed to the development of superior additional C-S-H by silica fume, thereby improving the porosity on the surface of RCA [55,56] due to its higher surface area, which results in denser microstructure and improved strength. This improved performance of the 10% silica fume is attributed to the better fineness of silica fume with better particle packing. This was even synthesised by the composite’s substantial cover, which augments the zone between the matrix and the coarse particles by the superior fineness of silica. Considering the effect of steel fibres, the compressive strength results of mixes with fibre were higher by 10 to 16% compared to their corresponding mixes without fibre.

### 3.3. Split Tensile Strength

**Figure 7:**
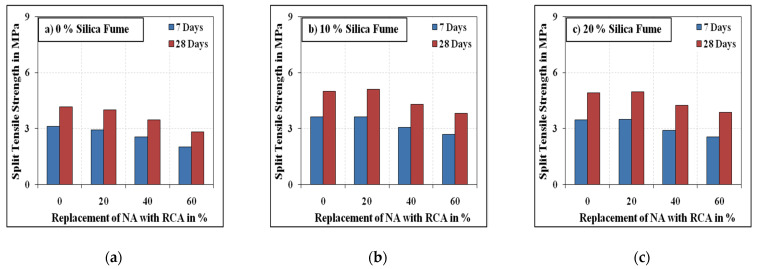
Non-fibrous specimens’ splitting tensile strength. (**a**) 0% Silica fume, (**b**) 10% Silica fume, (**c**) 20% Silica fume.

**Figure 8:**
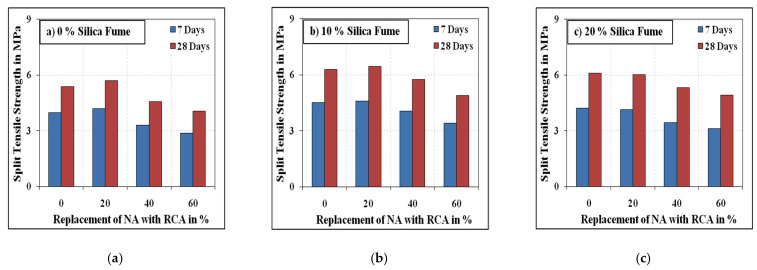
Fibrous specimens’ splitting tensile strength. (**a**) 0% Silica fume, (**b**) 10% Silica fume, (**c**) 20% Silica fume.

Figure 7 and Figure 8 summarise the splitting tensile strength results of all mixes. As for the compressive strength, the splitting tensile strength of the mixes decreased as the volume of RCA increased beyond 20%. The splitting tensile strength of the mixes with 20% RCA was approximately equivalent to that of reference specimens (0% RCA) with slight decrease or increase. On the other hand, the mixes with 40% and 60% RCA exhibited 9 to 19% and 20 to 35% respective strength reductions compared to the mixes without RCA. The reduction in the strength with the increasing amount of RCA is primarily on account of the availability of pores in RCA and the deprived matrix of the adhered mortar in RCA. The degradation of split strength of concrete with high replacement contents of RCA is attributed to the increasing number of weak links in concrete, which are formed because the thickness of ITZ between the RCA and binder paste is lesser than that of the ITZ developed between NA and binder paste [57,58]. Besides, the larger specific surface of the RCA due to its lesser size than the NA results in an increased number of ITZ leading to reduced strength properties of SCC [58]. Micro-hardness and microstructure are generally excellent in ITZ of RCA owing to its rough surface and pre-absorbed water [59]. Pozzolans remaining in the RCA may further consume CH and react to contribute to strength growth surrounding the ITZ [60].

Unlike the compressive strength, the inclusion of steel fibres increased the split tensile strength by 18 to 43% compared to the mixes without steel fibre. The split tensile strength values of the mixes with 10% silica fume were 9 to 35% higher than those of the corresponding mixes without silica fume. Having 20% of the contents be silica fume resulted in a maximum reduction of 15% in split strength compared to the mixes with 10% silica fume. Similarly to the compressive strength results, the split tensile strength results of the mixes with 20% silica fume were slightly higher than those of the mixes without silica fume.

### 3.4. Impact Resistance

**Figure 9:**
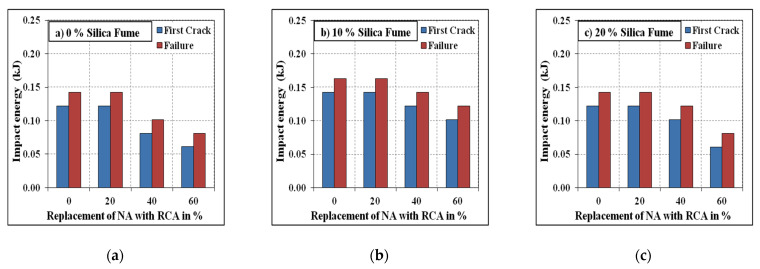
Non-fibrous specimens’ impact energy. (**a**) 0% Silica fume, (**b**) 10% Silica fume, (**c**) 20% Silica fume.

**Figure 10:**
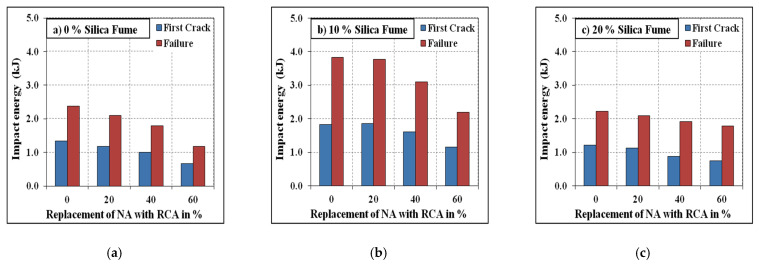
Fibrous specimens’ impact energy. (**a**) 0% Silica fume, (**b**) 10% Silica fume, (**c**) 20% Silica fume.

The resistance under impact of the developed SCC mixes was tested using the technique reported by ACI 544-2R [61]. The absorbed impact energy with varying replacement levels of NA with RCA for the mixes with and without steel fibres are shown in Figure 9 and Figure 10. It has been evident from the test results that the energy absorbed by the specimens under impact tends to reduce with the increasing quantum of RCA. The reason is that the availability of pores present in the adhered mortar and the weaker interfacial transition zone led to a decrease in the energy absorption capacity of the specimens. The specimens incorporating steel fibres were found to absorb higher impact energy compared to plain ones, which is directly influenced by the ductility nature of the specimens under the influence of steel fibres [62,63]. However, the specimens reinforced with steel fibres exhibited higher reductions in impact energy due to RCA compared to their corresponding plain specimens. This was due to the weaker interlocking mechanism between the fibres and the weakly adhered mortar present on the surface of RCA. The silica fume’s effect on the energy absorption capacity was similar to those on the compressive strength and split tensile strength, where the 10% replacement of silica fume resulted in the highest impact energies compared to the 0% and 20% replacement levels.

**Figure 11:**
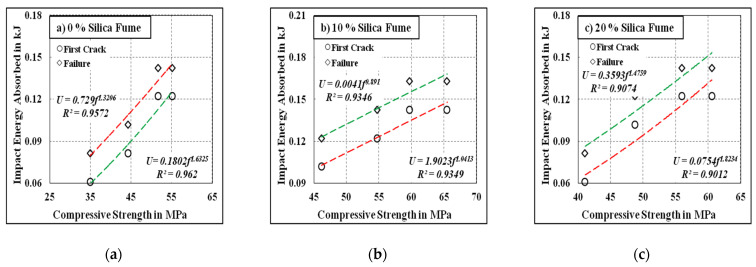
Compressive strength vs. Impact Energy for the non-fibrous specimens. (**a**) 0% Silica fume, (**b**) 10% Silica fume, (**c**) 20% Silica fume.

**Figure 12:**
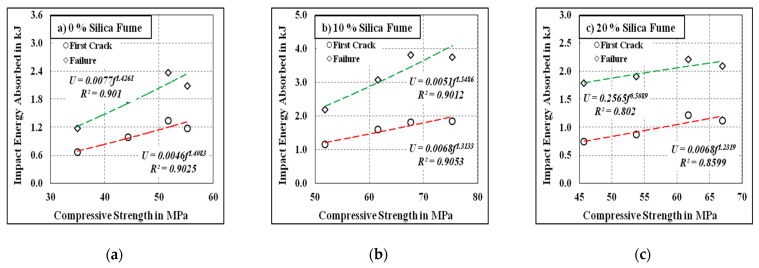
Compressive strength vs. impact energy for the fibrous specimens. (**a**) 0% Silica fume, (**b**) 10% Silica fume, (**c**) 20% Silica fume.

Figure 11 and Figure 12 depict the correlations among the developed mixes’ energy absorption capacities, and their corresponding compressive strength results with and without the inclusion of steel fibre, respectively. The correlations between compressive strength and energy absorption capacity under impact for the first crack and failure are clearly seen in the figures. The obtained R^2^ values were greater than 0.90 for all cases except for the fibrous mix comprising 20% silica fume, where the R^2^ values were 0.8599 and 0.802 for the first crack and failure, respectively. This might have been due to the incomplete pozzolanic reaction developed at a higher silica fume inclusion rate, which can be counteracted under elevated temperature curing.

**Figure 13:**
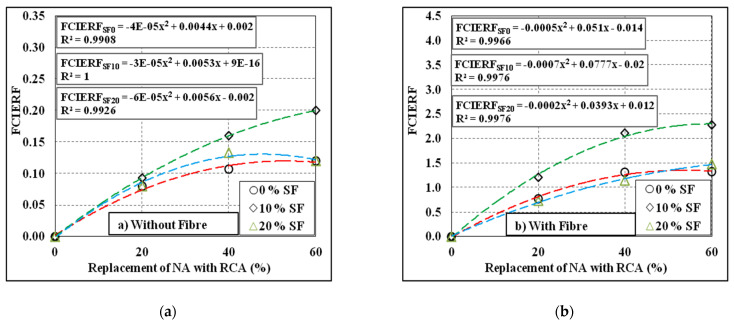
FCIERF results of the tested mixes. (**a**) Without fibre, (**b**) With fibre.

**Figure 14:**
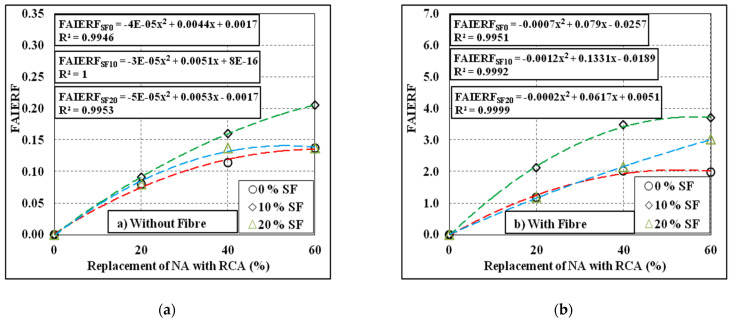
FAIERF results of the tested mixes. (**a**) Without fibre, (**b**) With fibre.

There are strong correlations among the first crack impact energy reduction factor (FCIERF) and the failure impact energy reduction factor (FAIERF), and the replacement levels of NA with RCA, inclusion of silica fume and inclusion of steel fibre, as shown in Figure 13 and Figure 14. A second-order polynomial trend line was used to establish the correlations of FCIERF and FAIERF with the quantity of RCA. The FCIERF and FAIERF were calculated using Equation (2). According to the statistics, it can be shown that FCIERF and FAIERF have high correlations with the varying levels of RCA, and the R^2^ values recorded were in general more than 0.99, which approves the accuracy of the equation introduced.

FCIERF/FAIERF = (U_ab_/U_00_) × (*w/b*) × (*ab*/100) (2)

where U_00_ is the impact energy in Joules for control the concrete mix, U_ab_ is the impact energy in Joules for RCA volume of “ab” and *w/b* is the water-binder ratio (constant = 0.40).

**Figure 15:**
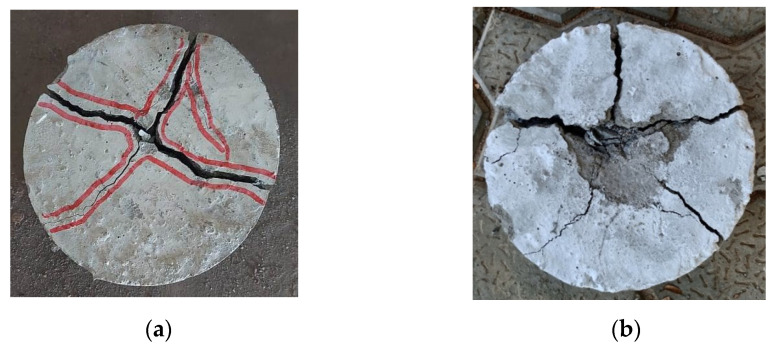
Typical failure patterns of the tested specimens under impact loading: (**a**) Non-fibrous specimen; (**b**) fibrous specimen.

Figure 15 illustrates the typical failure patterns of the specimens with and without steel fibres due to the impact loading application at the age of 28 days. It was observed from the failure patterns that the crack width was significantly smaller for specimens with higher volumes of silica fume, irrespective of the availability of steel fibres. The mixes with high volume replacements of NA with RCA exhibited wider cracks, which might have been due to the effortless delamination of the newly formed mortar from the adhered mortar available on the surface of RCA due to its inferior quality. The specimens without steel fibres tended to split into pieces at the stage of failure [64,65,66,67], whereas the specimens incorporating steel fibres failed without splitting [68,69,70,71]. This is attributed to the bridging mechanism of steel fibres with the concrete, which improved the interlocking between the particles in the matrix [72,73].

## 4. Conclusions

This research investigated the fresh and mechanical properties of SCC made of RCA, where RCA derived from C&D waste was treated with a magnesium sulphate solution of 10% concentration for a process duration of 5 days. The resulting aggregates were used as a partial replacement for NA (0%, 20%, 40% and 60%) in the production of SCC that incorporates partial replacement of cement with silica fume (0%, 10% and 20%). All SCC mixes incorporate GGBFS as a constant 20% replacement of cement with or without steel fibres. The fresh properties (density, slump flow, J-ring, T_500_ and V-funnel) and hardened properties (compressive strength, split tensile strength and impact resistance) of the developed mixes were assessed. From the experimental results obtained, the following conclusions can be drawn:

1. The fresh properties of the developed mixes showed a decreasing trend with the increases in silica fume and RCA and with the inclusion of steel fibres. Irrespective of the reductions, all mixes satisfied the minimum requirements for structural applications.
2. The compressive strength and split tensile strength of the mixes increased with the volume of silica fume up to 10%, though a subsequent decrease was recorded at 20%. However, the strengths of the mixes with 20% silica fume were slightly higher than those of the control mixes (0% silica fume).
3. Mixes with 20% RCA volume showed slightly improved strength properties over the control concrete mixes, though subsequent reductions were observed at higher replacement levels. The maximum reductions in the compressive strength and split tensile strength at 60% RCA were 34% and 35%, respectively, compared to the control concrete mixes.
4. The inclusion of steel fibres led to significant improvements in the strength and energy absorption characteristics of the developed SCC mixes under impact loading.
5. The physical properties of the treated RCA became increasingly inferior as the concentration of MgSO_4_ and the process duration rose. Therefore, RA treated with 10% MgSO_4_ concentration over a process duration of 5 days exhibited the best performance among the investigated ranges of treatment concentrations and durations. Moreover, SCC comprising 20% RCA and 10% silica fume exhibited the maximum compressive strength. As a result, the recommended RCA and silica fume replacement levels were 20 and 10%, respectively.
6. Featured Application: Treatment of RCA with MgSO_4_ solution does not have an environmental impact on disposing of the solution after treatment. In addition to the standard structural parts, RCA-based SCC can be used to produce very complicated and densely reinforced structural elements, which can eliminate the need to plan for harsh vibrations and positively affects the final quality.

## Figures and Tables

**Table 1:** Chemical compositions of the binders used in this investigation.

Concentration (%)	SiO_2_	Al_2_O_3_	Fe_2_O_3_	CaO	MgO	Na_2_O	K_2_O	SO_3_
OPC	21.8	6.60	4.10	60.1	2.10	0.40	0.45	2.20
GGBFS	30.97	17.41	1.03	36.77	9.01	0.69	0.46	1.82

**Table 2:** Mix proportioning of the tested mixes.

Series	Mix No.	Volume (kg/m^3^)								Steel Fibres (%)
		Cement	GGBFS	Silica Fume	NA 0–6	NA 6–16	RA 0–6	RA 6–16	Water	
Series 1	S1R0	420	172	0	1033	526	0	0	219	0
	S1R20	420	172	0	207	105	826	421	219	0
	S1R40	420	172	0	413	211	620	316	219	0
	S1R60	420	172	0	620	316	413	211	219	0
Series 2	S2R0F	420	172	0	1033	526	0	0	219	1
	S2R20F	420	172	0	207	105	826	421	219	1
	S2R40F	420	172	0	413	211	620	316	219	1
	S2R60F	420	172	0	620	316	413	211	219	1
Series 3	S3R0	361	172	59	1033	526	0	0	219	0
	S3R20	361	172	59	207	105	826	421	219	0
	S3R40	361	172	59	413	211	620	316	219	0
	S3R60	361	172	59	620	316	413	211	219	0
Series 4	S4R0F	361	172	59	1033	526	0	0	219	1
	S4R20F	361	172	59	207	105	826	421	219	1
	S4R40F	361	172	59	413	211	620	316	219	1
	S4R60F	361	172	59	620	316	413	211	219	1
Series 5	S5R0	302	172	118	1033	526	0	0	219	0
	S5R20	302	172	118	207	105	826	421	219	0
	S5R40	302	172	118	413	211	620	316	219	0
	S5R60	302	172	118	620	316	413	211	219	0
Series 6	S6R0F	302	172	118	1033	526	0	0	219	1
	S6R20F	302	172	118	207	105	826	421	219	1
	S6R40F	302	172	118	413	211	620	316	219	1
	S6R60F	302	172	118	620	316	413	211	219	1

**Table 3:** The recommended values for rheological properties of SCC as per EFNARC guidelines.

Test Method	Minimum-Maximum
Slump flow (mm)	650–800
T_500_ (S)	2–5
V-funnel (S)	6–12
J-Ring (mm)	0–10

**Table 4:** Fresh properties of the tested mixes.

Series	Mix No.	Density (kg/m^3^)	Slump Flow (mm)	T_500_ mm (s)	V-Funnel (s)	J-Ring (mm)
Series 1	S1R0	2401	751	2.4	7.8	7.1
	S1R20	2367	740	2.4	7.7	7.3
	S1R40	2356	721	2.7	8.2	7.7
	S1R60	2317	684	3.2	9.1	8.4
Series 2	S2R0F	2427	728	2.8	9.0	7.4
	S2R20F	2381	719	2.7	9.3	7.3
	S2R40F	2366	702	3.0	9.7	7.9
	S2R60F	2347	669	3.6	10.3	8.6
Series 3	S3R0	2425	772	2.6	7.7	7.5
	S3R20	2400	762	2.5	8.2	7.5
	S3R40	2366	731	2.8	8.6	7.8
	S3R60	2327	702	3.3	9.6	8.5
Series 4	S4R0F	2447	747	2.8	8.9	7.9
	S4R20F	2405	731	3.0	9.3	8.0
	S4R40F	2386	712	3.3	9.7	8.4
	S4R60F	2367	688	3.9	10.8	9.0
Series 5	S5R0	2468	757	2.4	8.0	7.0
	S5R20	2451	741	2.4	8.4	7.1
	S5R40	2379	704	2.8	9.1	7.5
	S5R60	2353	680	3.5	10.2	8.3
Series 6	S6R0F	2482	732	3.0	8.5	7.5
	S6R20F	2441	715	3.1	9.0	7.4
	S6R40F	2409	693	3.7	10.2	8.0
	S6R60F	2399	644	5.1	11.4	9.0

## Data Availability

Not applicable.
